# Neuroprotective effects of *Withania somnifera* in BPA induced-cognitive dysfunction and oxidative stress in mice

**DOI:** 10.1186/s12993-019-0160-4

**Published:** 2019-05-07

**Authors:** Hareram Birla, Chetan Keswani, Sachchida Nand Rai, Saumitra Sen Singh, Walia Zahra, Hagera Dilnashin, Aaina Singh Rathore, Surya Pratap Singh

**Affiliations:** Department of Biochemistry, Institute of Science, Banaras Hindu University, Varanasi, 221005 India

**Keywords:** Cognitive impairment, Neurotoxicity, Oxidative stress, Spatial memory, Reactive oxygen species, Biomarkers

## Abstract

**Background:**

Bisphenol A (BPA), a major endocrine disruptor and a xenobiotic compound is used abundantly in the production of polycarbonate plastics and epoxy resins. Human exposure to this compound is primarily via its leaching from the protective internal epoxy resin coatings of containers into the food and beverages. In addition, the plastics used in dental prostheses and sealants also contain considerable amount of BPA and have a high risk of human exposure. Since it is a well-known endocrine disruptor and closely mimics the molecular structure of human estrogen thereby impairing learning and memory. *Withania somnifera* (Ws), commonly known as Ashwagandha is known for its varied therapeutic uses in Ayurvedic system of medicine. The present study was undertaken to demonstrate the impairment induced by BPA on the spatial learning, working memory and its alleviation by Ws in Swiss albino mice. The study was conducted on thirty Swiss albino mice, randomly distributed among three groups: control, BPA and BPA + Ws. The behavioral recovery after treatment with Ws was investigated using the Y-maize and Morris water maize test. Whereas, for the estimation of recovery of NMDA receptor which is related to learning and memory in hippocampus region by western blot and immunohistochemistry. Furthermore, the oxidative stress and antioxidant level was assessed by biochemical tests like MDA, SOD and catalase.

**Results:**

The study revealed that administration of Ws alleviated the behavioral deficits induced by BPA. Alongside, Ws treatment reinstated the number of NMDA receptors in hippocampus region and showed anti-oxidative property while ameliorating the endogenous anti-oxidant level in the brain.

**Conclusion:**

These findings suggest that Ws significantly ameliorates the level of BPA intoxicated oxidative stress thereby potentially treating cognitive dysfunction which acts as the primary symptom in a number of neurodegenerative diseases.

## Background

Bisphenol A (BPA, 4zx, 40-isopropylidene-2-diphenol) is the building block in the manufacture of polycarbonate plastics, epoxy resins, compact discs, dental sealants and thermal papers [1]. The primary source of BPA exposure in human population, worldwide is due to its ubiquitous presence in food and beverage packaging [2]. The different routes of BPA exposure are oral, transdermal and through inhalation [3]. Previous studies have reported the presence of alarming levels of conjugated BPA in urine, serum, umbilical cord fluid and, most hazardously in breast milk [4, 5]. BPA has been identified as a causative agent for various perilous disorders such as cancer, thyroid deformities, cognitive impairments and male infertility as a result of its xenoestrogenic activity [6]. The surrounding environment has a major impact on health of the population consequently, the environmental exposure of endocrine disrupting chemicals cannot be ignored. The primary factor for cognitive impairment is ageing but there are various associated factors including the exposure of various toxins, pesticides, head injury, along with genetic predisposition [7]. BPA has been reported to cross the blood–brain barrier in different concentrations leading to various behavioral changes associated with cognitive impairment along with increased aggression, hyper-reactivity learning deficits, and increased drug dependency [8]. *N*-methyl-d-aspartate-receptors (NMDARs), a specific kind of ionotropic glutamate receptor found in the hippocampal region of the mid brain are crucial in controlling the synaptic plasticity and cognition [9]. The hippocampus is the main site for learning and memory. BPA has been reported to decrease the expression of the subunits of NMDAR along with estrogen receptor in the hippocampus [10].

In the past decade, researchers have been determined to investigate the role of *Withania somnifera* (Ws), a revered Indian Ayurvedic herb commonly known as Indian Ginseng or Indian Winter Cherry, for its medicinal properties [11–13]. Studies have evaluated the medicinal properties of Ws extracts in various diseases and have established the cognition promoting effects [13, 14], anxiolytic activity [15] and neuroprotective effects [16]. Studies have made it evident that Ws root extract not only significantly lowers lipid peroxidation but also augments superoxide dismutase (SOD) and catalase activities, thereby modulating the oxidative network in the cytosol [17]. Moreover, Ws has been documented to be an effective anti-stress, anti-inflammatory, and anti-aging medicinal herb [18].

The present study is based on assessing the effect of BPA in swiss albino mice and the effect of the treatment therapy of the root extract of Ws. Our aim was to investigate the neuroprotective role of Ws in memory enhancement after damage through BPA induced cognitive dysfunction in mice.

## Materials and methods

### Chemicals required

Acetic acid, disodium hydrogen phosphate, nicotinamide adenine dinucleotide reduced form (NADH), potassium chloride, phenazinemethosulphate, nitro-blue tetrazolium, disodium phosphate and sodium dihydrogen phosphates were procured from Sisco Research Laboratories (Mumbai, India). Reagents for Bradford assay were purchased from GeNei (Bengaluru, India). Hydrogen peroxide and potassium-dichromate were purchased from Merck (Darmstadt, Germany). Sodium dodecyl sulphate (SDS), thiobarbituric acid (TBA), trireagent, and zinc sulphate were procured from Sigma-Aldrich (St. Louis, MO, USA). Rabbit polyclonal to NMDAR2A (ab16646) antibody were procured from Abcam (place), mouse monoclonal anti-β-actin (sc47778) purchased from Santacruz (USA), biotinylated Goat polyclonal to Rabbit IgG and anti-mouse secondary antibodies, anti-goat AP conjugated and bovine anti-rabbit (AP) conjugated secondary antibodies were acquired from Santa Cruz Biotechnology (Santa Cruz, CA, USA). DABCO, anti-fading agent was purchased from Fluka analytical (Shanghai, China). Secondary antibody, GT × Rb IgG-Biotin conjugated (62111028001A) and GT × Ms IgG Biotin-conjugated (cat#105261) (GeNei, Bengaluru, India). Streptavidin-peroxidase, normal goat serum and DAB system was procured from Genei Pvt. India Ltd. Polyvinylidene difloride (PVDF) membranes were procured from Merck Millipore (USA).

### Medicinal plants and preparation of extracts

The plants of *W. somnifera* were procured from the Institute of Medical Science, Banaras Hindu University, Varanasi, India. Primarily, the roots of Ws were desiccated and crushed. About 600 g prepared powder of the Ws root was soaked overnight in 1000 mL ethanol and refluxed using a soxhlet apparatus. Further, the extract was concentrated under reduced pressure and stored at 4 °C. Subsequently, the prepared extract was firstly filtered using filter paper (Whatman No. 1) and later dried through evaporation in rotary vacuum evaporator under reduced pressure and temperature (below 40 °C). For in vivo assays, the extract was suspended in 0.7% carboxy methyl cellulose (CMC, S.D fine chemicals, India).

### Animal dosing

**Fig. 1:**
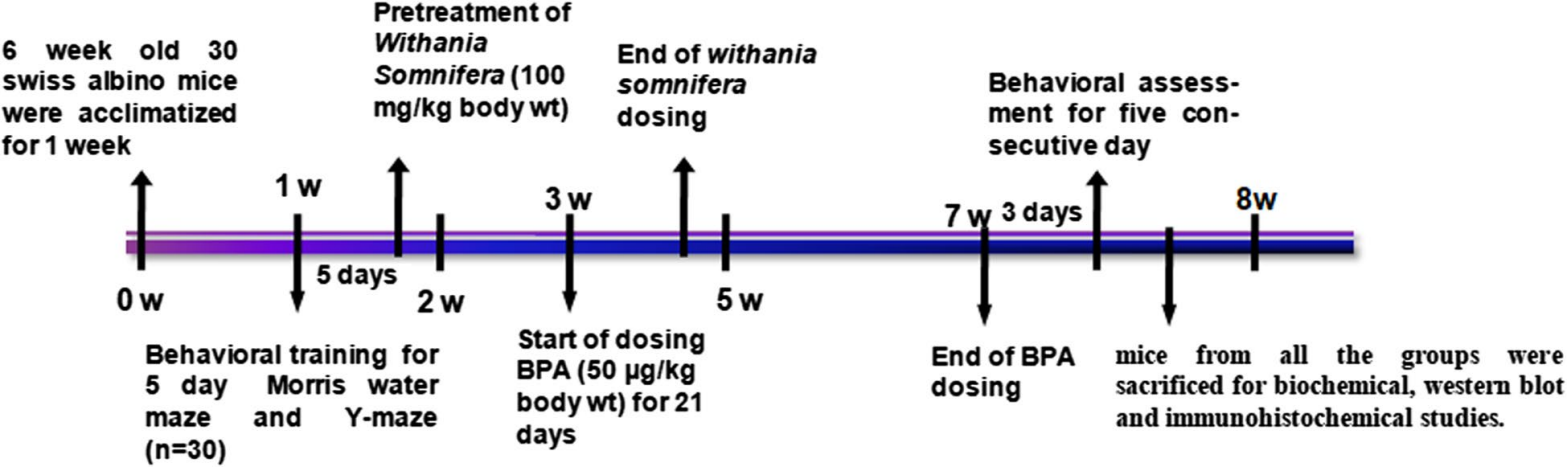
Research design and testing time scale. Time scale of complete experimental procedure

The experiments performed on animals in the laboratory were as per the norms of Institutional Ethics Committee. Male Swiss albino mice weighing 25 ± 5 g required for the experiments were provided by animal house of the Institute of Medical Science, Banaras Hindu University, Varanasi, India. The animals were kept in clean polypropylene cages in a maintained environment of temperature (22 ± 5 °C), humidity (45–55%) and 12 h light–dark cycle with proper access to the standard diet of mice pellet and water. Post acclimatization, they were separated into three groups, each group with ten mice. Group-I served as control with vehicle administration. Group-II was administered with BPA 50 µg/kg bw/day for 21 days. Group-III was intoxicated with BPA with given Ws root extract treatment (100 mg/kg bw/day, orally). Mice were pre-treated with Ws for 1 week. After the completion of dosing, mice were subjected to various tests to analyze their behavioral performance and later sacrificed for further tests (Fig. 1).

### Y-maze test

Y-maze is a spontaneous alternation behavioural test, used to assess behavioural tasks in preclinical study. It is based on the willingness of rodents for exploration of a completely new environment to understand their spatial learning and memory. The analysis occurs in a Y-shaped maze with three identical black arms (35 × 5 × 15 cm) at a 120° angle from each other. The mouse is put in the centre of the maze and allowed free access to all the three arms. Three trials were performed for each mouse where the test animal was placed in one of the three arms and allowed to move freely for 5 min in the direction of any arm. Every time a mouse chose an altered arm than the one it was placed on, it was termed as alteration and the number of these arm entries and alterations were recorded to calculate the percentage of alteration [19].

### Morris water maze test

The Morris water maze (MWM) behavioral procedure was established and standardized for evaluating the spatial learning of rodents. The basic structure of the MWM test involves a large circular black painted pool (100 cm^2^ in diameter and 55 cm in height) filled with water to a depth of 26 cm (23–25 °C). It is made as featureless as possible from inside. A target made of flexi-glass was submerged all through the experiment at fixed quadrant below 1 cm water level. The maze was divided into four equal quadrants (E, W, N, S). The test was always conducted at fixed time to avoid the effect of diurnal variation. The test for analyzing the effect of BPA in mice model was carried out for 5 consecutive days with two tests per day. Maximum latency time was set as 60 s. In case, the mouse was unable to locate the platform for a minute, it was directed towards the platform leaving it to rest for 15 s. On 7th day, a probe test was conducted by removing the platform and the time spent in the target quadrant was duly recorded. The escape latency time during test and time spent in target quadrant during probe trial for each mouse was analyzed [20–22].

### Biochemical analysis

#### Lipid peroxidation

Sample collection after the completion of behavioural tests, mice were euthanized and sacrificed by dislocation followed by decapitation to ensure minimum pain. The brain was removed and frozen immediately. Brain was dissected in ice cold conditions to isolate nigrostriatal tissue and it was stored at 80 °C until the biochemical analysis was performed. Level of lipid peroxidation was evaluated in accordance with standardized protocol with slight modifications in the hippocampus tissue of mice brain [23]. Furthermore, 10% of homogenate was taken and added with 10% SDS solution along with 20% acetic acid. Ultimately, 0.8% TBA was mixed and the final mixture was incubated in hot water (60 °C) bath for 60 min. Later, the mixture was cooled down and centrifuged. The supernatant was analyzed for absorbance at 532 nm with respect to control. Level of LPO was recorded in nmoles of MDA/mg protein.

#### Catalase and superoxide dismutase (SOD) activity

Rate of decomposition of hydrogen peroxide was evaluated through catalase activity using spectrophotometer [24]. Further, potassium dichromate and acetic acid (1:3) were added to the homogenized tissue and incubated in hot water bath (60 °C) for 10 min and absorbance was measured at 570 nm. The enzymatic activity was measured in moles/min/mg protein. While, for SOD assay, NADH was used as the substrate [25]. Absorbance was observed at 560 nm for both the tubes with reference to the blank. Absorbance of both the blank and test sample was compared to analyze the inhibition of Nitro blue tetrazolium chloride (NBT) reduction. This was also used for protein estimation. SOD can be defined as the amount of enzyme required for the inhibition of 50% NBT reduction at 560 nm in 1 min. SOD activity was expressed as unit/mg protein.

### Immunoblotting analysis

The hippocampus of male mice were used to prepare 5% homogenate in RIPA buffer [1× TBS, 1% nonyl phenoxypolyethoxylethanol-40, 0.1% sodium dodecyl sulfate, 0.6% sodium deoxycholate, 100 µg/ml phenylmethanesulfonyl fluoride and 5 µl/100 mg protease inhibitor cocktail]. Homogenate of the sample was prepared through centrifugation which was done at 12,000 rpm at 4 °C for about 15 min. The supernatant thus obtained was kept at − 20 °C for further use. From the homogenate, the amount of protein was assessed through Bradford method [26]. 35 µg of protein was taken, denatured and resolved in 10% Tris–glycine SDS-PAGE which was later transferred onto the nitrocellulose membrane (Millipore, USA). Blocking of the membrane was done for about 2 h with 7% (w/v) non-fat milk prepared in 1× TBS at room temperature. The blot was then incubated with primary antibodies (goat anti-NMDA, 1:1000; goat anti-β-actin, 1:500 at 4 °C) overnight. Washing was done thrice for 10 min each in 0.1% TBST (0.1% Tween-20 in 1× TBS) and was further incubated with secondary antibodies (GT × Rb IgG-Biotin conjugated (62111028001A) followed by washing thrice for 5 min each in 0.1% TBST and developed by DAB method [27, 28].

### Immunohistochemistry

Anesthesia was given to the mice of each group using diethyl ether and intracardial perfusion was done by injecting chilled 0.9% saline and 4% para-formaldehyde, made in 0.1 M phosphate buffered saline (PBS) of pH 7.4. After decapitation, the brains were isolated and stored in 10% para-formaldehyde for overnight and were transferred to 10%, 20% and 30% sucrose solutions, respectively. Using a cryomicrotome (Leica, Wetzlar, Germany), 20 µm thick coronal sections of hippocampus was cut. Then, immunohistochemistry of NMDA was done in hippocampus using the standard procedure with slight modifications [29, 30]. DABCO was used as the mounting media. By using Nikon fluorescent microscope (Thermo Fisher Scientific), the images were taken and by using Image J software (NIH, United States) the immunofluorescence was evaluated. The results were reported as % area value in hippocampus region of brain.

### Statistical analysis

By applying one-way analysis of variance (ANOVA) using Student–Newman–Keuls test in the graph pad prism 5.0, all the data were analyzed. Report of the values was done as mean ± SEM and *p* values < 0.05 were well thought-out as statistically significant. A repeated measures ANOVA was performed for MWM escape latency analysis and Y-maze.

## Results

**Fig. 2:**
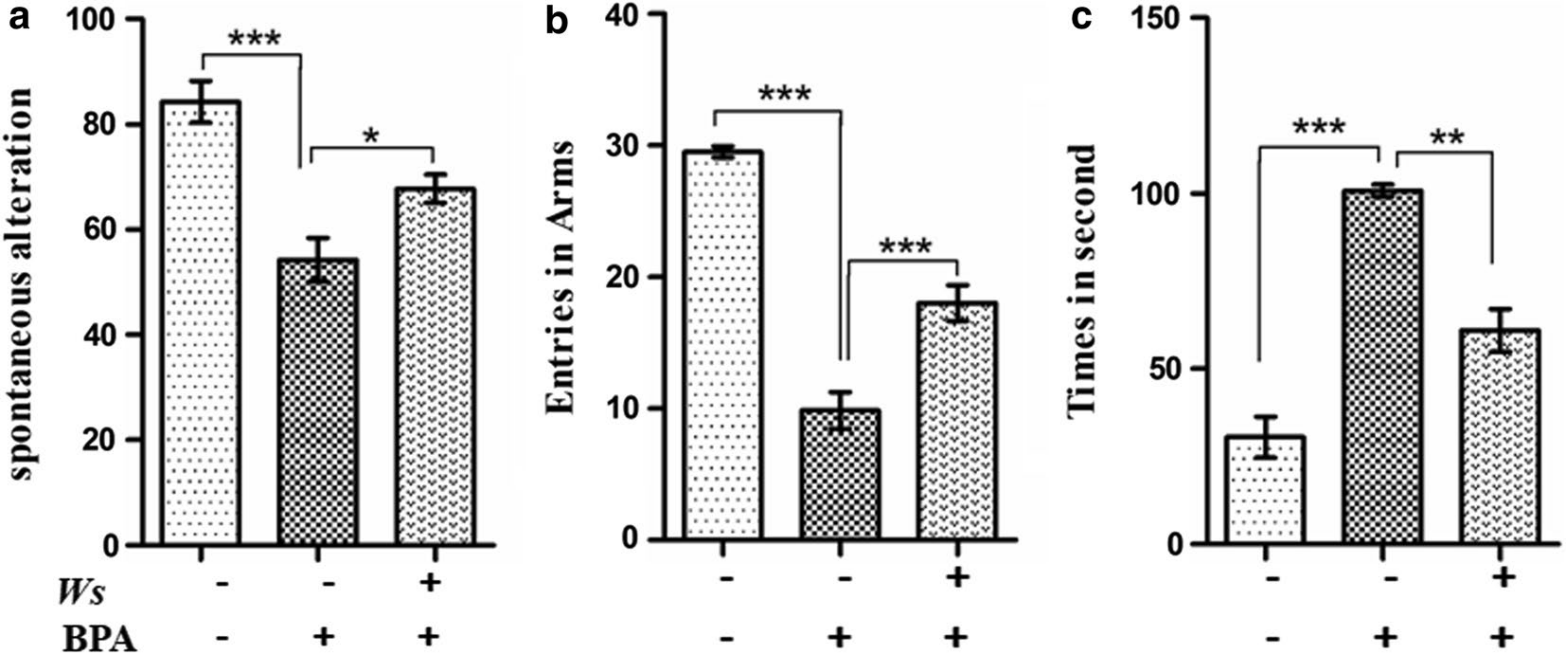
Effect of BPA and BPA + Ws on spatio-temporal memory. **a** Spontaneous alteration. **b** Entries in Arms and Morris water maze test to reach the hidden platform. **c** Escape latency (in seconds). Data is expressed in terms of mean ± SEM (n = 10). (*p < 0.05, **p < 0.01, ***p < 0.001). SEM: standard error of mean; CONT: control; Ws: *Withania somnifera*; BPA: bisphenol A; SEM: standard error of mean

In the Y-maze test, spontaneous alternation and arm entries of all mice were observed (above six entries within 5 min). The BPA mice group had a lesser total number of spontaneous alteration (p < 0.001) (Fig. 2a) and entries in arms (p < 0.001) (Fig. 2a), as compared to the control group. But in case Ws treatment both spontaneous alteration (p < 0.05) (Fig. 2a) and entries in arms (p < 0.001) (Fig. 2b) were greater than BPA intoxicated mice and Ws treated mice indicating greater alteration and exploratory locomotion. Thereby, suggesting impairment of BPA intoxicated mice in the measure of short term spatial memory.

### Morris water maze test

In MWM test, BPA exposed mice took longer time to reach the hidden platform as compared to that of control (p < 0.001) (Fig. 2c). While, the time taken by the Ws administered BPA mice group was considerably lesser as compared to the BPA-intoxicated (p < 0.01) group. After removing the platform at the end of experiment, it was observed through subsequent probe test that the BPA-treated group spent lesser time in the target quadrant and more time in other quadrants when compared to the control group and Ws group, respectively. Thus, the data obtained clearly indicated that exposure to BPA significantly impaired the spatial memory of mice while, Ws treatment improved it.

### Ws forestalls BPA induced oxidative stress and balances anti-oxidant levels

**Fig. 3:**
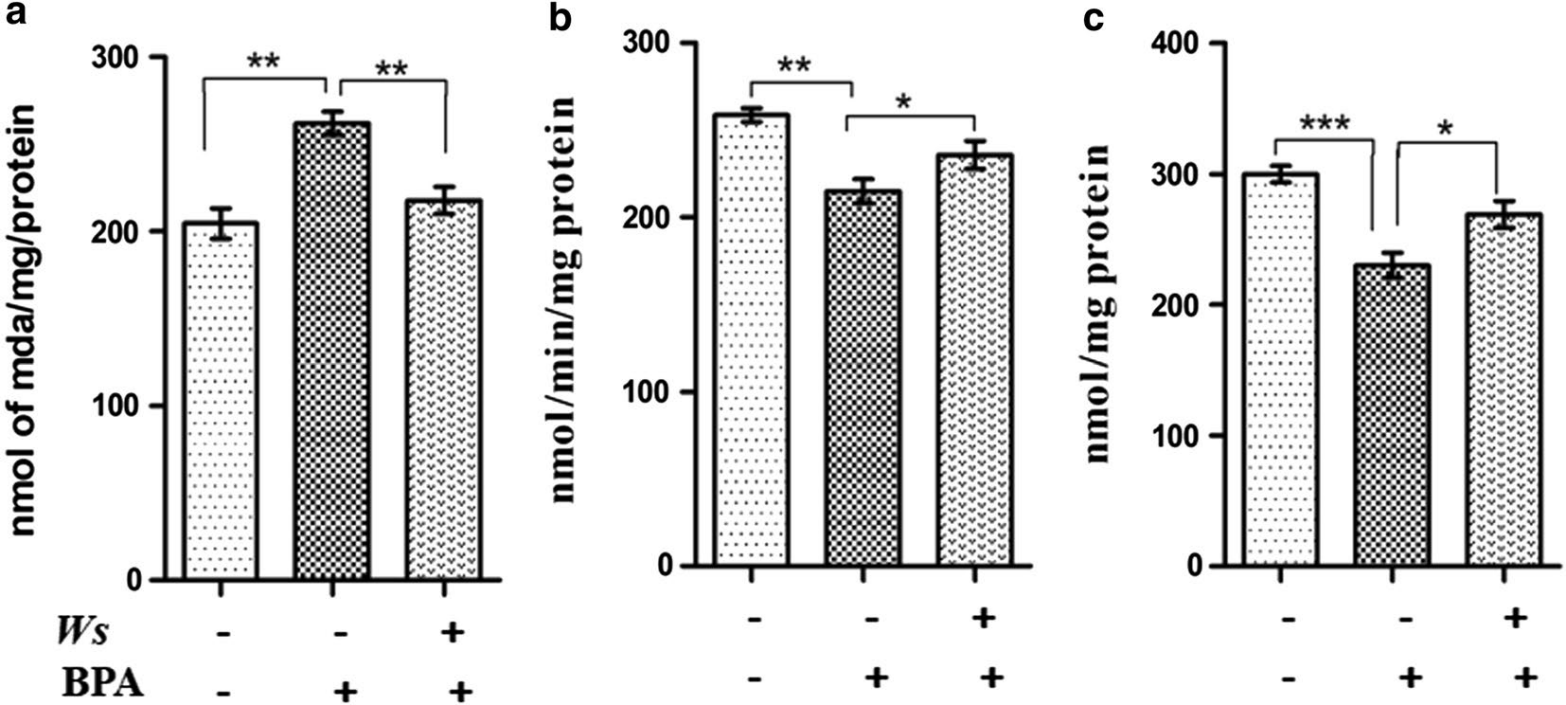
Biochemical estimation of oxidative stress markers in the Hippocampus region of mice brain. **a** MDA, **b** CAT, **c** SOD. Values are expressed as mean ± SEM (n = 6) (*p < 0.05, **p < 0.01, ***p < 0.001). MDA: malondialdehyde; SOD: superoxide dismutase; CAT: catalase; SEM: standard error of mean

Lipid-peroxidation level was assessed by analyzing the level of MDA and the effect of Ws on it was assessed in hippocampus (Fig. 3a). MDA level was found to be considerably increased in BPA-exposed impaired mice as compared to the control group in hippocampus region of brain (p < 0.01) (Fig. 3a). The treatment with Ws on the other hand decreased the MDA level in the BPA group (Fig. 3a). The reactive oxygen species (ROS) produced in the hippocampus was also efficiently scavenged by the anti-oxidative enzymes such as SOD and catalase. In our result, Ws was found to modulate the activity of antioxidant enzymes. The catalase level was significantly reduced (p < 0.01) in BPA group as compared to control group within hippocampus (Fig. 3b). Interestingly, Ws was found to significantly restore the levels of catalase in BPA intoxicated mice (p < 0.05) (Fig. 3b). While, in the case of BPA-exposed mice, activity of SOD was found to be significantly decreased (p < 0.001) as compared with that of control group (Fig. 3c). However, on Ws treatment, the activity of SOD was seen to be significantly elevated in comparison to BPA-exposed mice p < 0.05 (Fig. 3c).

### Western blot analysis of NMDA receptor

**Fig. 4:**
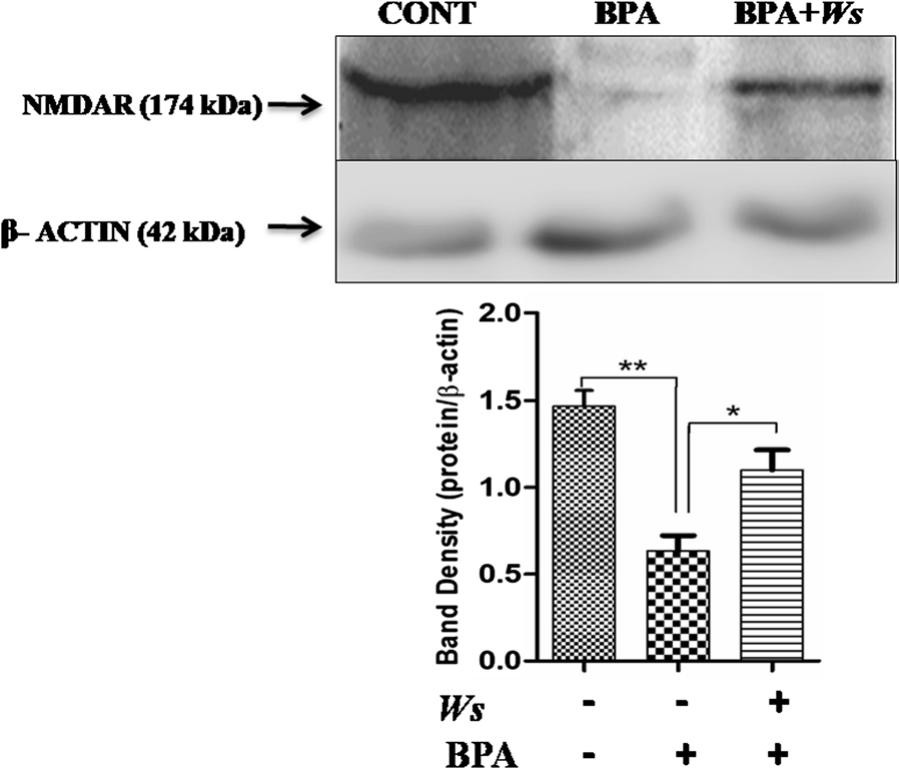
Expression level of NMDAR in Hippocampus region of mice brain. Expression of NMDAR (174 kDa), were determined by Western blot analysis. Values are expressed as mean ± SEM (n = 6) (*p < 0.05, **p < 0.01). β-Actin taken as endogenous control. NMDAR: *N*-methyl-d-aspartate-receptors; SEM: standard error of mean

After assessing the behavioural and biochemical parameters, the expression levels of NMDAR (147 kDa), (Fig. 4) were estimated using Western blots in tissue lysates isolated from hippocampus region. A reduced expression of NMDAR (p < 0.001) was observed in BPA-intoxicated mice as compared to the control group. In Ws treatment group increased NMDA level (p < 0.01) was observed when compared to BPA-intoxicated mice.

### Immunohistochemistry of NMDA receptor

**Fig. 5:**
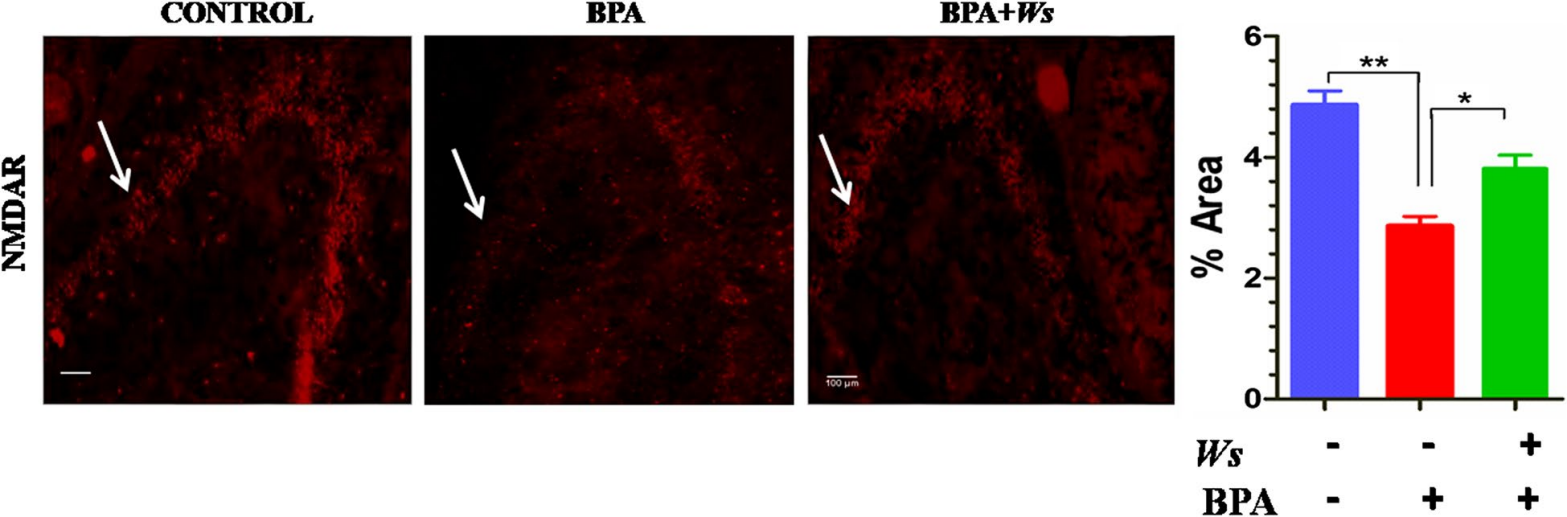
IHC-IF staining of NMDAR. With 20× magnifications after staining. Expression of NMDAR in Hippocampus of mice brain. Value expressed as mean ± SEM (n = 5) (*p < 0.05, **p < 0.01)

BPA administration resulted in significant loss of NMDA receptor (p < 0.01) neurons in Hippocampus as compared to normal saline treated control mice (Fig. 5). The treatment of Ws to BPA group, showed significant increase of NMDAR (p < 0.05) in comparison to BPA intoxicated mice.

## Discussion

The effect of BPA 50 µg/kg bw/day only and in combination with Ws (100 mg/kg bw/day) was investigated on the cognition of mice. Here we hypothesized to explore the neurotoxic effects of BPA on cognitive impairment and memory dysfunction and its alleviation using Ws root extract. BPA intoxication was found to severely damage the main site for learning and memory, i.e., hippocampus.

The use of Ws extract in treating several diseases is documented from the very beginning in Ayurveda, the Indian system of medicine [31]. It has been demonstrated that the antioxidant property of herbal medicines have a substantial role in curing neurodegenerative diseases [16, 32]. Withaferin A is the major bioactive compound present in Ws [33]. These naturally occurring compounds have the property to enhance the regeneration of neurons by enhancing the growth of axons and dendrites [34, 35]. The Ws extract has also been found to be effective against brain aging, beneficial for mental health and prevents motor impairment [36]. Withaferin A, has been reported to cure a number of neurological disorders [37–41], though very few studies have focused on the toxicity of Ws and Withaferin A. The LD50 value of 2000 mg/kg body wt for Ws root extract containing 4.5% Withaferin A and the safe dose has been reported upto 200 mg/kg body wt [42, 43].

The widely known endocrine disruptor, bisphenol A is found to be released by different polycarbonate plastics and food packaging [44]. Because of the huge availability of the toxicant in the environment, humans get frequently exposed to it on daily basis [2]. Interestingly, BPA has been found to increase oxidative stress in different regions of brain, thus the fact potentiates the role of BPA in various neurodegenerative disorders [45–47]. In rodents too, BPA is found to impair the cognition [48]. Experimentally, it has been proven that perinatal exposure of BPA interferes with the process of sexual differentiation and affects the behaviour of the newly-born animals [49]. Accordingly, Xu et al. [50] suggested that on perinatal exposure to BPA, both spatial and avoidance memory were affected, subsequently influencing the normal behavioural development from neonatal to adulthood. In the current study, the results indicate that intoxication of BPA for 21 days significantly impaired the cognitive function and increased the time to reach the platform in spatial navigation task when compared to the control group in Morris water maize test. However, when the administration of Ws was done, the learning and memory was found to be significantly enhanced. We also performed Y-maze test to study the willingness of rodents towards exploring the new environments was done using Y-maze spontaneous alternation test [51]. Generally, the animals like to visit the newer arm of the maze rather than wandering into the previous one. In our study, the level of alteration and the entries in the newer arm, both were concomitantly reduced by BPA intoxication, while it considerably enhanced on treatment of Ws in the BPA-intoxicated mice.

The cognitive impairment was seen to be linked with the hippocampal down-regulation of NMDA receptor [10, 52, 53]. In this regard, our result also demonstrated that BPA intoxication leads to decreased expression of NMDA receptor. Whereas, Ws administration is capable of restoring the level of NMDAR in BPA intoxicated mice. Moreover, the Ws extract was also effective in enhancing the learning and memory in BPA intoxicated mice.

Oxidative stress has been closely associated with the pathophysiology of various diseases such as aging, atherosclerosis, diabetes, cancer and other degenerative disorders [54, 55]. The endogenous anti-oxidant enzymes like catalase, SOD and other molecules are efficient in scavenging the amplified ROS generated in brain and other tissues [56]. Thus, the involvement of oxidative stress in BPA induced cognitive impairment was assessed using various parameters like catalase, SOD and LPO in the hippocampal region of mice. Increased level of MDA, which subsequently marks increased LPO level, was observed in the hippocampus of BPA intoxicated mice as compared to control. This result was in accordance with various studies which demonstrated that on the exposure of BPA, there is an increased generation of ROS in the brain, decreased endogenous antioxidants in the liver and the epididymal sperm [57–59]. Furthermore, Aydogan et al. [60] also suggested that enhanced level of MDA in the brain was observed upon exposure to BPA. In the present study, significant impairment in learning and memory was observed in BPA intoxicated mice in comparison to the control. Hence, this fact can be put forward that sustained oxidative stress is one of the possible phenomena responsible for cognitive impairment in BPA administered mice group. Antioxidants play a major role in preventing the progression of degenerative diseases by alleviating the oxidative stress [16]. Researchers have suggested the alteration in the redox potential in experimental animals upon contamination with the environmental insults [61]. Various reports have suggested the neuroprotective effects of Ws through its antioxidant nature in the brain [62–64]. The ethanolic extract of the roots of Ws in our study was seen to significantly reduce the level of LPO and increase the activities of SOD and catalase through its free radical scavenging activity. Thus, Ws acts as a potent anti-oxidant, regulating the level of endogenous antioxidants, which are usually depleted as a result of aggravated oxidative stress. Furthermore, it has been seen to provide protection against oxidative damage and memory impairment induced by BPA.

## Conclusion

Dementia and cognitive impairment has been seen to severely affect the quality of life and life span of elderly and are the primary symptoms of neurodegenerative disorders. Moreover, oxidative stress plays an essential role in the pathogenesis of these diseases and is linked to cognitive impairment. BPA is a potent endocrine disruptor and is seen to induce the cognitive impairment. The present study deals with the neuroprotective activity of Ws against BPA-induced oxidative stress and memory impairment in mice. Therefore, our study put-forward Ws as potent drug candidate for BPA-induced cognitive impairment.

## Data Availability

The datasets used and/or analyzed during the current study are available from the corresponding author on reasonable request.

## Abbreviations

SOD: superoxide dismutase
MDA: malondialdehyde
BPA: bisphenol A
Ws: *Withania somnifera*
NMDAR: *N*-methyl-d-aspartate-receptor
MWM: Morris water maze
